# Antibiotic use for acute respiratory tract infections (ARTI) in primary care; what factors affect prescribing and why is it important? A narrative review

**DOI:** 10.1007/s11845-018-1774-5

**Published:** 2018-03-12

**Authors:** Ray O’Connor, Jane O’Doherty, Andrew O’Regan, Colum Dunne

**Affiliations:** Graduate Entry Medical School, University of Limerick, Limerick City, Limerick, 000 Ireland

**Keywords:** Adult or paediatric, Antibacterial agent, Antibiotic prescription, Patient expectations, Upper respiratory tract infection

## Abstract

**Background:**

Antimicrobial resistance is an emerging global threat to health and is associated with increased consumption of antibiotics. Seventy-four per cent of antibiotic prescribing takes place in primary care. Much of this is for inappropriate treatment of acute respiratory tract infections.

**Aims:**

To review the published literature pertaining to antibiotic prescribing in order to identify and understand the factors that affect primary care providers’ prescribing decisions.

**Methods:**

Six online databases were searched for relevant paper using agreed criteria. One hundred ninety-five papers were retrieved, and 139 were included in this review.

**Results:**

Primary care providers are highly influenced to prescribe by patient expectation for antibiotics, clinical uncertainty and workload induced time pressures. Strategies proven to reduce such inappropriate prescribing include appropriately aimed multifaceted educational interventions for primary care providers, mass media educational campaigns aimed at healthcare professionals and the public, use of good communication skills in the consultation, use of delayed prescriptions especially when accompanied by written information, point of care testing and, probably, longer less pressurised consultations. Delayed prescriptions also facilitate focused personalised patient education.

**Conclusion:**

There is an emerging consensus in the literature regarding strategies proven to reduce antibiotic consumption for acute respiratory tract infections. The widespread adoption of these strategies in primary care is imperative.

## Introduction

Antimicrobial or antibiotic resistance (AMR) is an increasingly serious threat to global public health [1]. Consequently, there is an emerging risk that standard antibiotic treatments no longer work making infections harder or impossible to control [2]. Increasing consumption of antibiotics is associated with the development of antibiotic resistance at individual, community, country and regional levels [3–7]. It is estimated that 25,000 humans in the EU die annually as a result of infections caused by resistant bacteria, at a societal cost of approximately €1.5 billion annually [8].

The rate of use of antibiotics in Ireland for quarter 4 of 2016 was 23 defined daily doses (DDD) per 1000 inhabitants per day, and this is up from 20 DDD per 1000 inhabitants per day in 2009 [9]. In comparison to other EU countries, antibiotic use in Ireland is mid-range [9]. The Scientific Advisory Committee of the Irish National Disease Surveillance Centre (NDSC) was tasked in 2001 to produce a strategy document in response to the growing problem of antimicrobial resistance. This resulted in the “Strategy for the control of Antimicrobial Resistance in Ireland” (SARI) [10], which produced a number of national guidelines and advised the Irish Health Services Executive (HSE) on matters relating to the prevention and control of antimicrobial resistance and healthcare-associated infection. The work of SARI has more recently been taken over by the National Clinical Programme for the prevention of healthcare-associated infection (HCAI) and antimicrobial resistance (AMR) under the auspices of the HSE. This has led to HSE guidelines issued for antibiotic prescribing in primary care [11].

Over the last 30 years, no major new types of antibiotics have been developed [12]. This in combination with increasing AMR means that we are dealing with a finite and diminishing antibiotic resource. Therefore, prudent antibiotic stewardship programmes, aiming to ensure the judicious use of antimicrobials by preventing their unnecessary use, have been established [1, 10, 13–16].

Acute respiratory tract infection (ARTI), which incorporates the term “upper respiratory infection” (URTI), is the most common reason for antibiotic prescription in adults, and these prescriptions are often inappropriate [17]. The benefits of antibiotics are marginal for the management of most cases of ARTI [18–25], including sore throat [26, 27]. With few exceptions [28], inappropriate prescribing of antibiotics for patients with mainly URTI is common [29–33]. It is estimated that 75% of overall antibiotic prescribing takes place in primary care [34]. Large variations in antibiotic prescribing for URTI exist and are difficult to explain [22, 35]. Some potential explanations include the fact that many general practitioners (GPs) do not think that antibiotic prescribing in primary care is responsible for the development of antibiotic resistance [36–40] and, on average, acute cough can last from nine [41] to 18 days [42], while public expectation is for a duration of 7–9 days [42].

This paper reviews the literature on factors affecting antibiotic prescribing for ARTI in primary care. We consider specifically the effects of patient expectation and desire for antibiotics to treat respiratory symptoms, other patient characteristics, primary care provider (PCP) characteristics and the setting of the consultation. We also review the evidence behind current strategies employed to address this public health challenge.

## Methods

### Definitions

Where not otherwise specified, the term *primary care provider* (PCP) refers to all healthcare professionals dealing with the public in the primary care setting including GPs and non-medical professionals such as nurse practitioners, practice nurses, maternal child health nurses and pharmacists. Where specific studies mention particular types of PCP, this is indicated.

### Search strategy

While this article is not intended to be a systematic review, a comprehensive search of the literature was performed through Cochrane Library, Embase, PubMed Central, Scopus, Medline and CINAHL, looking at English language journals from 1997 to date. Original studies were included. Review papers such as editorials, opinion pieces, studies from secondary care, case reports, articles written prior to 1997 and studies involving lower respiratory tract infections only were excluded. The search terms used were “Respiratory tract infection” or “upper respiratory tract infection”, “antibiotic” or “antibacterial agents”, “patient expectations” or “patient attitudes”, antibiotic* and prescri*, and “upper respiratory infection” or “upper respiratory tract infection” or “upper respiratory infection (URI) in children” or “upper respiratory infection (URI) in adults or adolescents”. Duplicates were excluded during this process, and bibliographies were screened by two of the authors (JOD ROC) for further relevant papers. The search strategy used is outlined in detail in Table 1.

**Table 1:** Search strategy

Search string 1	
“Respiratory tract infection” or “upper respiratory tract infection” or “antibiotic” or “antibacterial agents” or “patient expectations” or “patient attitudes”	
Database	Results based on inclusion criteria
Scopus	49
Search string 2	
(“upper respiratory tract infection” OR urti) AND (patient AND expectation) AND (antibiotic* AND prescri*))	
Database	Results based on inclusion criteria
Scopus	20
Search string 3	
“patient expectations” and “antibiotics” or “antibacterial agents” and “upper respiratory infection” or “upper respiratory tract infection” or “upper respiratory infection (URI) in children” or “upper respiratory infection (URI) in adults or adolescents”	
Database	
Medline and CINAHL	22
Search string 4	
((“respiratory tract infections”[MeSH Terms] OR (“respiratory”[All Fields] AND “tract”[All Fields] AND “infections”[All Fields]) OR “respiratory tract infections”[All Fields] OR (“upper”[All Fields] AND “respiratory”[All Fields] AND “tract”[All Fields] AND “infection”[All Fields]) OR “upper respiratory tract infection”[All Fields]) AND (“anti-bacterial agents”[All Fields] OR “anti-bacterial agents”[MeSH Terms] OR (“anti-bacterial”[All Fields] AND “agents”[All Fields]) OR “anti-bacterial agents”[All Fields] OR “antibiotic”[All Fields])) AND ((“patients”[MeSH Terms] OR “patients”[All Fields] OR “patient”[All Fields]) AND expectation[All Fields]) AND (“2007/06/12”[PDat]: “2017/06/08”[PDat])	
PubMed	57
Search string 5	
“patient expectations” and “antibiotics” or “antibacterial agents” and “upper respiratory infection” or “upper respiratory tract infection” or “upper respiratory infection (URI) in children” or “upper respiratory infection (URI) in adults or adolescents**”.**	
Database	
EBSCO	22
Search string 6	
“patient expectation” AND “upper respiratory infection” AND “antibiotics” AND (“upper respiratory tract infection” OR urti) patient AND expectation) AND TITLE-ABS-KEY (antibiotic* AND prescri*)	
Database	
Cochrane	34
Search string 7	
“respiratory tract infections”[MeSH Terms] OR (“respiratory”[All Fields] AND “tract”[All Fields] AND “infections”[All Fields]) OR “respiratory tract infections”[All Fields] OR (“upper”[All Fields] AND “respiratory”[All Fields] AND “tract”[All Fields] AND “infection”[All Fields]) OR “upper respiratory tract infection”[All Fields]	
Database	
PubMed	57

## Results

The effect that different factors play in the PCP’s decision to prescribe antibiotics to treat acute respiratory infections may be categorised as follows.

### Primary care provider factors

#### Time constraints

GPs and other PCPs working in highly pressurised clinical environments managing high patient volumes are more likely to prescribe antibiotics for ARTI [29, 38, 43–45]. The level of antibiotic prescriptions issued increased in line with numbers of patients seen per day, resulting in shorter consultations [29, 45]. Suggested reasons for this excess antibiotic prescribing were lack of time in the consultation to discuss management alternatives and to inform the patients about the poor efficacy of antibiotics [38, 43, 44].

#### Primary care providers’ perceptions of patients’ expectation for antibiotics

GPs and other primary care doctors are more likely to prescribe antibiotics to patients who expect them or whom they believe expect them [36, 40, 41, 43, 44, 46–56]. This experience is replicated with other non-medical PCPs [44]. Patient expectation has been described as an all-encompassing term that is affected by factors such as limited time in the consultation, diagnostic uncertainty and poor doctor patient communication [36]. High prescribers were concerned about patient satisfaction and were unaware that they differed from their peers [56].

#### Primary care providers’ personal factors

A Canadian analysis prescribing for patients aged 66 years or older with non-bacterial ARTIs showed that primary care physicians who were in mid or late career or who were seeing high patient volumes, or who were trained outside of Canada or the USA were more likely to prescribe antibiotics [29]. However, the physician rationale for prescribing was not studied. A systematic review of studies from both ambulatory care and hospital settings concluded that inadequate knowledge and misconceptions of prescribing are prevalent among physicians from the USA, the UK and Peru, with pocket-sized guidelines seen as an important source of information [40]. Such misconceptions included prescribing antibiotics for purulent nasal discharge and thinking that occasional use of narrow spectrum antibiotics had a negligible effect on AMR [40]. A single cross-sectional study looking at 5937 ARTI visits to 102 primary care physicians in Canada found no association between empathy or burnout and antibiotic prescribing for ARIs in primary care [57].

Based on this, we can deduce that doctors’ professional training, their career stage and time pressures can be seen as important factors affecting their decision to prescribe antibiotic for ARTI (Table 2).

**Table 2:** Factors influencing antibiotic prescription

Factor	Characteristic increasing antibiotic prescriptions	Characteristic decreasing antibiotic prescriptions	Neutral or uncertain effect on antibiotic prescriptions
PCP characteristics	PCP’s time constraints		
	PCPs’ perceptions of patients’ expectation for antibiotics		
	Mid or late career physicians		
	Physicians seeing high patient volumes		
	Physicians trained outside of Canada or USA		
Patient factors Interventions to improve antibiotic prescriptions	High patient expectation	Low patient expectation	
	Socioeconomic background: Deprived Fee paying	Less deprived but not fee paying	
		Mass media interventions targeting the public	Single educational interventions
		Multifaceted educational campaigns targeting PCPs and the public	Passively leaving educational material in the waiting room
		Multifaceted educational interventions in general practice	
		Delayed Prescriptions	
Communication skills		PCP training in communication skills	
Clinical factors	Diagnostic uncertainty	Point of care testing including CRP and procalcitonin	
	Perceived severity of illness		
Social and system factors	Daycare providers		
	Direct patient access to antibiotics		
			Out of hours service

*PCP* primary care provider, *CRP* C-reactive protein

### Patient factors

#### Patient expectation

Measurement of patient expectation for antibiotic treatment for ARTI varies from 74% [58, 59] to 10% [41], with many measurements in between [60–64]. The study showing 10% patient expectation for antibiotics was conducted in China among patients presenting with ARTI symptoms and found that concern about illness severity and obtaining symptomatic treatment were the main reasons for consulting with ARTI rather than obtaining antibiotics [41]. The two studies showing the highest patient expectation rates were both studies of parents’ attitudes to antibiotic prescribing for their children who were not sick at the time, and were based in Greece and Palestine. Patient satisfaction varies with antibiotic prescription policies for ARTI and patients were less satisfied in practices with low antibiotic prescribing rates, and a cautious approach to antibiotic prescribing may require a trade-off in terms of patient satisfaction [41, 65]. Patients were also less satisfied when they expected but were not prescribed antibiotics [48, 66, 67]. However, receiving information and reassurance from the HCP was also associated with high patient satisfaction (Table 3) [64, 68].

**Table 3:** List of studies included in this review

Year	Author	Title of paper	Study type	Location
2016	Abad et al.	Prescription strategies in acute uncomplicated respiratory infections. A randomised clinical trial	Randomised controlled trial; primary care	Spain
2013	Ackerman et al.	One size does not fit all: evaluating an intervention to reduce antibiotic prescribing for acute bronchitis	Telephone cross-sectional survey; primary care	USA
2013	Agnew et al.	Delayed prescribing of antibiotics for respiratory tract infections: use of information leaflets	Intervention trial; primary care	Ireland
2016	AHRQ	Improving antibiotic prescribing for uncomplicated acute respiratory tract infections	Systematic review; all settings	USA
2004	Altiner et al.	Acute cough: a qualitative analysis of how GPs manage the consultation when patients explicitly or implicitly expect antibiotic prescriptions	Qualitative study; primary care	Germany
2013	Angoulvant et al.	Randomised controlled trial of parent therapeutic education on antibiotics to improve parent satisfaction and attitudes in a paediatric emergency department	Randomised controlled trial; emergency department tertiary paediatric hospital	France
2015	Anthierens et al.	Clinicians’ views and experiences of interventions to enhance the quality of antibiotic prescribing for acute respiratory tract infections	Qualitative study; primary care	Belgium, England, The Netherlands, Poland, Spain, Wales
2002	Arroll et al.	Do delayed prescriptions reduce the use of antibiotics for the common cold? A single-blind controlled trial	Randomised controlled trial; primary care	New Zealand
2014	Aryee et al.	Antimicrobial stewardship; can we afford to do without it?	Systematic review; all settings	Global
2016	Ashworth et al	Antibiotic prescribing and patient satisfaction in primary care in England: cross-sectional analysis of national patient survey data and prescribing data	Cross-sectional analysis; primary care	England
2014	Bell et al.	A systematic review and meta-analysis of the effects of antibiotic consumption on antibiotic resistance	Systematic review and Meta-analysis; all settings	Global
2017	Biezen et al.	Management of respiratory tract infections in young children—a qualitative study of primary care providers’ perspectives	Qualitative study; primary care	Australia
2012	Brookes-Howell et al.	Understanding variation in primary medical care: a nine-country qualitative study of clinicians’ accounts of the nonclinical factors that shape antibiotic prescribing decisions for lower respiratory tract infection	Qualitative study; primary care	Spain Italy England Wales Poland Hungary Norway The Netherlands Belgium
2009	Butler et al.	Variation in antibiotic prescribing and its impact on recovery in patients with acute cough in primary care: prospective study in 13 countries	Cross-sectional observational study; primary care	Spain Italy England Wales Poland Hungary Norway The Netherlands Belgium Sweden Finland Germany Slovakia
2014	Cabral et al.	How communication affects prescription decisions in consultations for acute illness in children: a systematic review and meta-ethnography	Systematic review; primary care paediatrics	Global
2016	Cabral et al.	Influence of clinical communication on parents’ antibiotic expectations for children with respiratory tract infections	Qualitative study; primary care; paediatrics	England
2016	Chaintarli et al.	Impact of a United Kingdom-wide campaign to tackle antimicrobial resistance on self-reported knowledge and behaviour change	Online cross-sectional survey; healthcare professionals and members of the public	UK
2013	Coenen et al.	Are patient views about antibiotics related to clinician perceptions, management and outcome? A multi-country study in outpatients with acute cough	Prospective observational study; primary care	Spain Italy England Wales Poland Hungary Norway The Netherlands Belgium Sweden Finland Germany Slovakia
2017	Courtenay et al.	Antibiotics for acute respiratory tract infections: a mixed-methods study of patient experiences of non-medical prescriber management	Mixed methods; cross-sectional and qualitative; primary care	UK
2015	Coxeter et al.	Interventions to facilitate shared decision making to address antibiotics use for acute respiratory infections in primary care (review)	Systematic review (CDSR); primary care	Global
2017	Debets et al.	Antibiotic prescribing during office hours and out-of-hours: a comparison of quality and quantity in primary care in the Netherlands	Retrospective review of patient data; primary care	The Netherlands
2014	Dempsey et al.	Primary care clinicians’ perceptions about antibiotic prescribing for acute bronchitis: a qualitative study	Qualitative semi structured interviews; primary care	Boston, USA
2001	Dowell et al.	A randomised controlled trial of delayed antibiotic prescribing as a strategy for managing uncomplicated respiratory tract infection in primary care	Randomised controlled trial; primary care	UK
2013	Ebell et al.	How long does a cough last? Comparing patients’ expectations with data from a systematic review of the literature	Quantitative community based survey and systematic review all settings	Georgia, USA
2003	Edwards et al.	Patients’ responses to delayed antibiotic prescription for acute upper respiratory tract infections	Quantitative questionnaire; community based	UK
2017	Elias et al.	Guideline recommendations and antimicrobial resistance: the need for a change	Scoping review; all settings	Global
1998	Fahey et al.	Systematic review of the treatment of upper respiratory tract infection	Systematic review of randomised controlled trials; all settings	Global
2008	Filipetto et al.	Patient knowledge and perception of upper respiratory infections, antibiotic indications and resistance	Quantitative questionnaire; community based	New Jersey, USA
2005	Fischer et al.	Influence of patient symptoms and physical findings on general practitioners’ treatment of respiratory tract infections: a direct observation study	Qualitative patient observation; primary care	Germany
2016	Fletcher-Lartey et al.	Why do general practitioners prescribe antibiotics for upper respiratory tract infections to meet patient expectations: a mixed methods study	Quantitative cross-sectional survey; primary care	Australia
2013	Francis et al.	Parents’ and clinicians’ views of an interactive booklet about respiratory tract infections in children: a qualitative process evaluation of the EQUIP randomised controlled trial	Qualitative semi-structured interviews; primary care	UK
2012	Francis et al.	Delayed antibiotic prescribing and associated antibiotic consumption in adults with acute cough	Observational study; primary care	Europe
2009	Francis et al.	Effect of using an interactive booklet about childhood respiratory tract infections in primary care consultations on reconsulting and antibiotic prescribing: a cluster randomised controlled trial	Pragmatic cluster randomised controlled trial; primary care	UK
2016	Gaarslev et al.	A mixed methods study to understand patient expectations for antibiotics for an upper respiratory tract infection	Mixed methods; population based	Australia
2002	Gambarelli et al.	Antibiotics in viral upper respiratory tract infections	Prospective observational study; primary care	Italy
2012	Garbutt et al.	Amoxicillin for acute rhinosinusitis: a randomised controlled trial	Randomised controlled trial; primary care	USA
2013	Gjelstad et al.	Improving antibiotic prescribing in acute respiratory tract infections: cluster randomised trial from Norwegian general practice (prescription peer academic detailing (Rx-PAD) study)	Randomised controlled trial; primary care	Norway
2004	Gonzales et al.	Antibiotic treatment of acute respiratory tract infections in the elderly: effect of a multidimensional educational intervention	Prospective non-randomised controlled trial; primary care	USA
2006	Goossens et al.	National campaigns to improve antibiotic use	Systematic literature review; primary and secondary care	Belgium France
2013	Grover et al.	Addressing antibiotic use for acute respiratory tract infections in an academic family medicine practice	Prospective clinical trial of educational intervention of HCPs and public; primary care	USA
2016	Gulliford et al.	Safety of reduced antibiotic prescribing for self limiting respiratory tract infections in primary care: cohort study using electronic health records	Cohort study; primary care	UK
2001	Haltiwanger et al.	Antibiotic-seeking behavior in college students: what do they really expect?	Cross-sectional study; college student population	USA
1996	Hamm et al.	Antibiotics and respiratory infections: are patients more satisfied when expectations are met?	Cross-sectional study; patients attending primary care	USA
2013	Harbarth et al.	Antimicrobial resistance: one world, one fight!	Background review document	Global
2013	Hardy-Holbrook et al.	Antibiotic resistance and prescribing in Australia: current attitudes and practice of GPs	Cross-sectional study of GPs; primary care	Australia
2003	Harris et al.	Optimising antibiotic prescribing for acute respiratory tract infections in an urban urgent care clinic	Controlled trial of educational intervention; primary care	USA
2016	Harris et al.	Appropriate antibiotic use for acute respiratory tract infection in adults: advice for high-value care from the American College of Physicians and the Centers for Disease Control and Prevention	Narrative literature review; all settings	Global
2010	Hoye et al.	Delayed prescribing for upper respiratory tract infections: a qualitative study of GPs’ views and experiences	Qualitative study; general practitioners	Norway
2011	Hoye et al.	Use and feasibility of delayed prescribing for respiratory tract infections: a questionnaire survey	Cross-sectional questionnaire study; general practitioners	Norway
2016	HPRA	Health Products Regulatory Authority report on antimicrobial resistance	Background review document	Global
2013	HPSC	Antibiotic consumption in the community in Ireland	Background review document	Ireland
2017	HPSC	Antibiotic use in Europe to Q4, 2016	Background review document	Europe
2017	HSE	List of conditions and preferred antimicrobial prescribing in primary care	Background review document	Ireland
2016	Hu et al.	Interventions to reduce childhood antibiotic prescribing for upper respiratory infections: systematic review and meta-analysis	Systematic review and meta analysis; paediatrics; all settings	Global
2010	Huttner et al.	Characteristics and outcomes of public campaigns aimed at improving the use of antibiotics in outpatients in high-income countries	Systematic review; all settings	Global
2003	Kallestrupp et al.	Parents’ beliefs and expectations when presenting with a febrile child at an out-of-hours general practice clinic	Cross-sectional study; primary care	Denmark
2015	Kautz-Freimuth et al.	Parental views on acute otitis media (AOM) and its therapy in children—results of an exploratory survey in German childcare facilities	Cross-sectional study; community based	Germany
2011	Kavenagh et al.	A pilot study of the use of near-patient C-Reactive Protein testing in the treatment of adult respiratory tract infections in one Irish general practice	Cross-sectional study; primary care	Ireland
2003	Kumar et al.	Why do general practitioners prescribe antibiotics for sore throat? Grounded theory interview study	Qualitative study; primary care	UK
2007	Lambert et al.	Can mass media campaigns change antimicrobial prescribing? A regional evaluation study	Retrospective controlled before–after study; population based	UK
2008	Lee et al.	Determinants of appropriate antibiotic use in the community—a survey in Sydney and Hong Kong	Cross-sectional study; population based	Hong Kong Australia
2013	Leydon et al.	A qualitative study of GP, NP and patient views about the use of rapid streptococcal antigen detection tests (RADTs) in primary care: ‘swamped with sore throats?’	Qualitative study; primary care	UK
2017	Lindberg et al.	Antibiotic prescribing for acute respiratory tract infections in Norwegian primary care out-of-hours service	Retrospective study; primary care	Norway
2002	Linder et al.	Blackwell Publishing Ltd. Desire for antibiotics and antibiotic prescribing for adults with upper respiratory tract infections	Prospective observational study; primary care	USA
1997	Little et al	Reattendance and complications in a randomised trial of prescribing strategies for sore throat: the medicalising effect of prescribing antibiotics	Randomised trial; primary care	UK
1997	Little et al.	Open randomised trial of prescribing strategies in managing sore throat	Randomised trial; primary care	UK
2002	Little et al.	Antibiotic prescribing and admissions with major suppurative complications of respiratory tract infections: a data linkage study	Data linkage study; primary and secondary care	UK
2005	Little et al.	Information leaflet and antibiotic prescribing strategies for acute lower respiratory tract infection: a randomised controlled trial	Randomised controlled trial; primary care	UK
2014	Little et al.	Delayed antibiotic prescribing strategies for respiratory tract infections in primary care: pragmatic, factorial, randomised controlled trial	Randomised controlled trial; primary care	UK
2013	Little et al.	Effects of internet-based training on antibiotic prescribing rates for acute respiratory-tract infections: a multinational, cluster, randomised, factorial, controlled trial	Cluster, randomised, factorial, controlled trial; primary care	Spain England Wales Poland The Netherlands Belgium
2013	Llor et al.	Low request of antibiotics from patients with respiratory tract infections in six countries: results from the Happy Audit Study	Prospective non-randomised study; primary care	Spain Denmark Sweden Lithuania Russia Argentina
2015	Mangione–Smith et al.	Communication practices and antibiotic use for acute respiratory tract infections in children	Cross-sectional study; primary and secondary care paediatrics	USA
2004	Martin et al.	Back-up antibiotic prescriptions could reduce unnecessary antibiotic use in rhinosinusitis	Cross-sectional study; community based	USA
2017	McDermott et al.	Qualitative interview study of antibiotics and self-management strategies for respiratory infections in primary care	Qualitative study; primary care	UK
2011	McKay et al.	Evaluation of the do bugs need drugs? Program in British Columbia: can we curb antibiotic prescribing?	Prospective observational study; primary care and community based	Canada
2016	McKay et al.	Systematic review of factors associated with antibiotic prescribing for respiratory tract infections	Systematic review; all settings	Global
2013	McNulty et al.	Expectations for consultations and antibiotics for respiratory tract infection in primary care: the RTI clinical iceberg	Two-phase qualitative and quantitative study; community based	England
2017	Mehta et al.	Antibiotic prescribing in patients with self-reported sore throat	Prospective cohort study; population based	UK
2013	Meropol et al.	Risks and benefits associated with antibiotic use for acute respiratory infections: a cohort study	Cohort study; primary care	UK
2014	Mohan et al.	Societal and physician perspectives on sinonasal diagnosis and treatment	Cross-sectional survey; community based, primary care, and ear, nose, and throat specialists	USA
2009	Moore et al.	Effect of antibiotic prescribing strategies and an information leaflet on longer-term reconsultation for acute lower respiratory tract infection	Balanced factorial randomised trial; primary care	UK
2017	Moore et al.	Influence of the duration of penicillin prescriptions on outcomes for acute sore throat in adults: the DESCARTE prospective cohort study in UK general practice	Prospective cohort study; primary care	UK
2017	Moore et al.	Symptom response to antibiotic prescribing strategies in acute sore throat in adults: the DESCARTE prospective cohort study in UK general practice	Prospective cohort study; primary care	UK
2011	Morgan et al.	Non-prescription antimicrobial use worldwide: a systematic review	Systematic review; community based	Global
2011	Murphy et al.	Influence of patient payment on antibiotic prescribing in Irish general practice: a cohort study	Cohort study; primary care (general practitioners)	Ireland
2012	Murphy et al.	Antibiotic prescribing in primary care, adherence to guidelines and unnecessary prescribing—an Irish perspective	Cohort study; primary care (general practitioners)	Ireland
2014	Nathan et al.	Antibiotic resistance—problems, progress, and prospects	Background review paper	Global
2015	NICE	Self-limiting respiratory tract infections—antibiotic prescribing overview	Background review paper	UK
2008	NICE	Respiratory tract infections (selflimiting): prescribing antibiotics	Background review paper	UK
2013	NICE	Acute cough—public expectation of symptom duration differs from published evidence	Background review paper	UK
2015	NICE	Antimicrobial stewardship: systems and processes for effective antimicrobial medicine use	Background review paper	UK
2017	NICE	Antimicrobial stewardship: changing risk related behaviours in the general population	Background review paper	UK
2015	O’Brien et al	Clinical predictors of antibiotic prescribing for acutely ill children in primary care: an observational study	Prospective observational study; general practice	Wales
2007	Ong et al.	Antibiotic use for emergency department patients with upper respiratory infections: prescribing practices, patient expectations, and patient satisfaction	Prospective observational study; emergency departments in secondary care	USA
2016	O’Sullivan et al.	Written information for patients (or parents of child patients) to reduce the use of antibiotics for acute respiratory tract infections in primary care	Systematic review; primary care	Global
2011	Panagacou et al.	Antibiotic use for upper respiratory tract infections in children: a cross-sectional survey of knowledge, attitudes, and practices (KAP) of parents in Greece	Cross-sectional study; community based	Greece
2017	Patel et al.	Understanding physician treatment decisions for the management of upper respiratory tract infections	Qualitative study; primary care physicians	USA
2011	Peters et al.	Managing self-limiting respiratory tract infections**:** a qualitative study of the usefulness of the delayed prescribing strategy	Qualitative study; primary care	UK
2015	Public Health England	Health matters: antimicrobial resistance	Background review paper	UK
2016	Rahman et al.	Antibiotic prescribing in public and private practice: a cross-sectional study in primary care clinics in Malaysia	Cross-sectional study; primary care	Malaysia
2017	Rebnord et al.	Factors predicting antibiotic prescription and referral to hospital for children with respiratory symptoms: secondary analysis of a randomised controlled study at out-of-hours services in primary care	Randomised controlled trial; primary care	UK
2015	Md Rezal et al.	Physicians’ knowledge, perceptions and behaviour towards antibiotic prescribing: a systematic review of the literature	Systematic review; all settings	Global
2017	Ritchie et al.	Previous antibiotic-related adverse drug reactions do not reduce expectations for antibiotic treatment of upper respiratory tract infections	Cross-sectional study; hospital inpatients	New Zealand
2014	Rooshenas et al.	The influence of children’s day care on antibiotic seeking: a mixed methods study	Mixed methods: cross-sectional study and qualitative interviews; community based	Wales
2011	Rousounidis et al.	Descriptive study on parents’ knowledge, attitudes and practices on antibiotic use and misuse in children with upper respiratory tract infections in Cyprus.	Cross-sectional study; community based	Cyprus
2016	Ryves et al.	Understanding the delayed prescribing of antibiotics for respiratory tract infection in primary care: a qualitative analysis	Qualitative study; general practitioners	England
2009	Sabuncu et al.	Significant reduction of antibiotic use in the community after a nationwide campaign in France, 2002–2007	Cross-sectional computerised database analysis pre and post intervention; community based	France
2012	Salazar et al.	Caregivers’ baseline understanding and expectations of antibiotic use for their children	Cross-sectional study; community based	USA
2009	SARI Hospital Antimicrobial Stewardship Working Group	Guidelines for Antimicrobial Stewardship in Hospitals in Ireland	Background review document	Ireland
2015	Schroeck et al.	Factors associated with antibiotic misuse in outpatient treatment for upper respiratory tract infections	Retrospective chart review; community based	USA
2013	Spinks et al.	Antibiotics for sore throat (review)	Systematic review (Cochrane); all settings	Global
2003	Vinker et al.	The knowledge and expectations of parents about the role of antibiotic treatment in upper respiratory tract infection—a survey among parents attending the primary physician with their sick child	Cross-sectional study; primary care	Israel
2015	Ru′n Sigurardo’ttir et al.	Appropriateness of antibiotic prescribing for upper respiratory tract infections in general practice: comparison between Denmark and Iceland	Cross-sectional study; primary care	Denmark Iceland
2017	Silverman et al.	Antibiotic prescribing for nonbacterial acute upper respiratory infections in elderly persons	Retrospective analysis of linked administrative healthcare data; primary care	Canada
2005	Soma et al.	Patients’ expectations of antibiotics for acute respiratory tract infections	Cross-sectional study; emergency department	Norway
2013	Spinks et al.	Antibiotics for sore throat	Systematic review; all settings	Global
2007	Spurling et al.	Delayed antibiotics for respiratory infections	Systematic review; all settings	Global
2016	Strandberg et al.	Interacting factors associated with low antibiotic prescribing for respiratory tract infections in primary healthcare—a mixed methods study in Sweden	Mixed methods; primary care	Sweden
2017	Sun et al.	Empathy, burnout, and antibiotic prescribing for acute respiratory infections: a cross-sectional primary care study in the US	Cross-sectional study; primary care	USA
2005	Taylor et al.	Effectiveness of a parental educational intervention in reducing antibiotic use in children: a randomised controlled trial	Randomised controlled trial; primary care paediatricians	USA
2014	Teng et al.	Antibiotic prescribing for upper respiratory tract infections in the Asia-Pacific region: a brief review	Narrative literature review; primary care	Asia-Pacific Region
2011	Tonkin-Crine et al.	Antibiotic prescribing for acute respiratory tract infections in primary care: a systematic review and meta-ethnography	Systematic review; primary care	Global
2017	Tonkin-Crine et al.	Clinician-targeted interventions to influence antibiotic prescribing behaviour for acute respiratory infections in primary care: an overview of systematic reviews	Systematic review; primary care	Global
2003	Vanden Eng et al.	Consumer attitudes and use of antibiotics	Cross-sectional study; community based	USA
2015	Vaz et al.	Prevalence of parental misconceptions about antibiotic use	Cross-sectional study; community based	USA
2013	Vinnard et al.	Effectiveness of interventions in reducing antibiotic use for upper respiratory infections in ambulatory care practices	Cross-sectional study pre and post intervention; primary care	USA
2013	Vodicka et al.	Reducing antibiotic prescribing for children with respiratory tract infections in primary care: a systematic review	Systematic review; primary care	Global
2004	Welschen et al.	Antibiotics for acute respiratory tract symptoms: patients’ expectations, GPs’ management and patient satisfaction	Cross-sectional study; primary care	The Netherlands
2014	WHO	Antimicrobial resistance; global report on surveillance	Background review document	Global
2008	Wigton et al.	How do community practitioners decide whether to prescribe antibiotics for acute respiratory tract infections?	Paper case vignette study; community practitioners	USA
2017	Wong et al.	Help-seeking and antibiotic prescribing for acute cough in a Chinese primary care population: a prospective multicentre observational study	Prospective multicentre observational study; primary care	China
2011	Wood et al.	A multi-country qualitative study of clinicians’ and patients’ views on point of care tests for lower respiratory tract infection	Qualitative study; primary care clinicians and adult patients	Spain Italy England Wales Poland Hungary Norway The Netherlands Belgium
2015	Yaeger et al.	Roles of clinician, patient, and community characteristics in the management of paediatric upper respiratory tract infections	Electronic health record study; community based	USA
2014	Zeng et al.	Systematic review of evidence-based guidelines on medication therapy for upper respiratory tract infection in children with AGREE instrument	Systematic review; all settings	Global
2015	Zyoud et al.	Parental knowledge, attitudes and practices regarding antibiotic use for acute upper respiratory tract infections in children: a cross-sectional study in Palestine	Cross-sectional study; primary healthcare	Palestine

*USA* United States of America, *UK* United Kingdom

Doctors may overestimate the pressure to prescribe antibiotics for acute cough [68–70] or other acute respiratory illnesses [50], often prescribing antibiotics for patients who did not request them [71]. There is mounting evidence that patients’ expectations for antibiotics for ARTI have lessened in recent years, especially where the consultation is more patient centred [41, 67, 72–76]. This illustrates the importance of a patient-centred consultation with good communication skills employed by the PCP.

#### Patient socioeconomic background

Patients with lower education level or who come from more deprived socioeconomic backgrounds who are likely to have less knowledge and understanding of the concept of antimicrobial resistance are more liable to be prescribed antibiotics for ARTI [38, 47, 77–79]. However, studies from Ireland, China and Malaysia have shown that patients paying a consultation fee are also more likely to receive antibiotics for ARTI because the clinicians are reluctant to see the patient “go away empty handed” [41, 80, 81].

### The effects of interventions to improve antibiotic prescriptions for ARTI

#### Educational interventions for patients

Mass media interventions such as national TV advertising campaigns in Belgium and France [82] and repeated mass media campaigns in France and England [83, 84] have been shown to reduce antibiotic prescribing for ARTI. However, these strategies work best when targeting both healthcare professionals and the public in mass media campaigns [85, 86]. Single educational interventions of leaflets or leaflet/videotape mailed to patients had little effect on reducing antibiotic prescribing rates for elderly [87] or paediatric [88] populations. Also, passively leaving this literature in the waiting room was found to have no effect [89].

#### Educational interventions for GPs and other PCPs

Multifaceted educational interventions in general practice including visits by peer academics, regional 1-day seminars, internet-based training in communication skills and C-reactive protein (CRP) testing, all aiming to reduce antibiotic prescription rates for ARTI and to reduce the use of broad-spectrum antibiotics, have been shown to be effective (90–92). GPs may need further guidance on how to answer the concerns of patients without interpreting these questions as a demand for antibiotics, as well as educating the patient about antimicrobial resistance and supporting a good patient–practitioner relationship [93]. This educational process is hindered by the fact that guidelines issued for GPs vary considerably regarding categorisation of evidence and recommendations [94], taking little account of local antimicrobial resistance patterns in their recommendations [95].

#### Educational interventions for PCPs and public

Antibiotic use for adults diagnosed with ARTI can be reduced using a combination of PCP and patient educational interventions [85, 86, 96, 97]. One such campaign based in Britain, Antibiotic Guardian, increased commitment to tackling AMR in both PCPs and members of the public, increased self-reported knowledge and changed self-reported behaviour particularly among people with prior AMR awareness [98]. In paediatric practice, a systematic review concluded that educational interventions targeting clinicians and parents of affected children are more effective than those for either group alone, and the most effective strategies address patient–clinician communication [99].

#### Delayed prescriptions

A well-documented strategy for reducing antibiotic prescriptions for ARTI is the use of delayed prescriptions. These are valid prescriptions issued at the time of the consultation. The PCP usually negotiates with the patient that they are not to be used immediately but only if the patient feels that their symptoms deteriorate or do not improve as expected [93]. There is substantial evidence that the use of delayed prescriptions has been associated with reduced antibiotic use [48, 100–108]. The DESCARTE study has been looking at the symptomatic outcome of acute sore throat in a random sample of 2876 adults according to antibiotic prescription strategy in routine care. It concludes that in the routine care of adults with sore throat, a delayed antibiotic strategy confers similar symptomatic benefits to immediate antibiotics [109]. Another paper from the same study also concluded that a small advantage in terms of reduced re-consultation for a 10-day course of penicillin could not be ruled out, but the effect is likely to be small [110]. However, a prospective observational cohort study of 14 primary care networks in 13 countries found the strategy to be unhelpful in reducing antibiotic consumption [111]. A qualitative study of GPs, trainee GPs and nurse prescribers found that issuing delayed prescriptions was not considered to be a helpful strategy for managing patients with self-limiting respiratory tract infections within primary care [112]. A Cochrane systematic review concluded that delayed prescriptions reduced patient satisfaction in some trials, which seems to have little advantage over avoiding them altogether where it is safe to do so [104]. In many cases, patients are happy to receive delayed prescriptions for antibiotics for ARTI [102–114]. A recent qualitative study of patients concluded that delayed prescribing is acceptable no matter how the delay is operationalised, but explanation of the rationale is needed by the PCP [114]. However, not all GPs issue delayed prescriptions [115] and not all patients may be content to receive them as they felt less enabled by consultations which resulted in delayed prescriptions [100]. In summary, delayed prescriptions may be a useful adjunct for PCPs in giving focused education to the patient about the expected natural history of their ARTI and what symptoms and signs to look out for that might indicate deterioration. Patient-focused education combined with the use of educational leaflets or booklets has been shown to reduce antibiotic consumption in children and adults [116–119]. The success of these strategies could depend on the level of communication skills of the PCP [116–119].

### The effect of communication skills of PCP including information delivered during the consultation

#### PCP–patient communication

Poor doctor–patient communication has been implicated in inappropriate antibiotic prescribing [36]. Significantly, explanation of long-term negative sequela does not appear to be a sufficiently strong incentive for patients and, consequently, antibiotic resistance needs to be explained as a more immediate health issue [61]. The need for, and effect of, such communication can be impressive. In a US study of 98 patients visiting family medicine clinical sites, whereas more than half the respondents recognised that treatment for colds did not require antibiotics, 70% erroneously indicated that viruses require antibiotic treatment and 95% of patients reported satisfaction when advised by their physician that antibiotic treatment was not necessary, even if they initially thought they needed antibiotics [120].

Training of PCPs in communication skills has been shown to reduce antibiotic prescribing for ARTI [92]. The results are even better when combined with point of care testing (POCT) [121]. A systematic review of the effectiveness of primary care-based interventions to reduce antibiotic prescribing for children with RTIs and a study involving structured interviews of parents of acutely ill children attending an out of hours service both indicate that clinical and communication skills in the PCP where they take a good history, examine the patient appropriately and give a good explanation of the cause of the illness help to improve appropriate antibiotic prescribing in paediatric practice [72, 89]. Also in paediatric consultations for ARTIs, parents receiving combined positive (e.g. measures to reduce fever and pain) and negative (e.g. recommendation against need for antibiotics) treatment recommendations were more likely to give the highest possible visit rating, which may reduce the risk of antibiotic prescribing [122]. When the doctors’ explanation is backed up by use of an information booklet, this reduced the number of antibiotics children consumed [116–118] without affecting parent satisfaction or numbers of return visits [118]. Such a booklet has high acceptability for both parents and clinicians [117]. Reductions in antibiotic prescribing for adults with ARTI have also been shown by the use of an information booklet [119].

The substance of what is communicated is also important. One study suggests that within-consultation communication aimed at reducing antibiotic expectations would be more effective if it is acknowledged that viral illness can be severe, thus validating the patient’s decision to attend (e.g. viral pneumonia) and that bacterial infections can be self-limiting and therefore may not need an antibiotic [123]. It also suggests that clearer explanations of the symptoms and signs of a child’s illness that indicate when antibiotics are and are not warranted would help reduce misunderstandings, as would reducing antibiotic prescribing that is not supported by evidence [123]. However, such communication which gives the patient more influence over the decision whether or not to prescribe an antibiotic may result in a variable outcome depending on the factors imposed by the healthcare system. A nine-country qualitative study described clinicians’ accounts of the non-clinical factors that shape antibiotic prescribing decisions for lower respiratory tract infection [124]. It showed that PCPs in specific primary care networks in Europe report that their prescribing decisions are influenced by factors imposed by the healthcare system, including direct patient access to antibiotics, systems to reduce patient expectations for antibiotics and lack of consistent treatment guidelines [124].

A systematic review has shown that misunderstandings have occurred because parents’ expressions of concern or requests for additional information were sometimes perceived as a challenge to the clinicians’ diagnosis or treatment decision and may be an important contribution to the unnecessary and unwanted prescribing of antibiotics [125]. Two systematic reviews concluded that interventions that aim to facilitate shared decision-making such as enhanced communication skills and patient information leaflets reduce antibiotic prescribing in primary care in the short term [126, 127]. Effects on longer-term rates of prescribing are uncertain, and it is unclear how any sustained reduction in antibiotic prescribing affects hospital admission, pneumonia and death [126].

### Clinical factors

#### Diagnostic uncertainty

Diagnostic uncertainty and fear of complications among the attending physicians is a common cause for prescribing antibiotics for ARTI [36, 51, 54]. A systematic review suggested that interventions which reduce uncertainty about appropriate ARTI management in primary care are likely to be effective in promoting prudent antibiotic use while remaining attractive to GPs and feasible in practice [74]. Point of care testing, discussed below, is also useful.

#### Perceived severity of illness

Perceived severity of the illness and abnormal results on clinical examination predicted increased antibiotic prescription [41, 55, 128–133]. Such prescribing may well be appropriate.

#### Point of care testing

Point of care testing (POCT) including CRP and procalcitonin has been shown to reduce antibiotic prescribing in primary care [121, 127, 134]. Their use is acceptable to patients [134–136] although clinicians have expressed concerns about using them in the consultation [136].

### Social and system factors

#### Out of hours prescribing of antibiotics

The suggestion that OOH antibiotic prescribing quality is worse than in daily practice does not seem founded as the higher OOH prescribing rates could be explained by a different population of presenting patients [137]. In Norway, antibiotic prescribing for ARTIs in OOH services is at the same level as in normal working hours, but with a higher prescription rate of penicillin V (PcV), which was close to the national goal of 80% proportion of PcV for ARTIs [45]. A suggested explanation was that doctors working in transparent out of hours units are more adherent to guidelines than doctors working in regular general practice are. Antibiotic prescribing increased during busy sessions [45].

#### Influence of daycare providers

Some daycare providers encourage parents of children with infections to consult general practice and seek antibiotics [138]. Parents’ perceptions of daycare providers’ requirements may override their own beliefs of when it is appropriate to consult and seek antibiotic treatment [138]. This is a potentially harmful attitude, which should be challenged.

#### Direct patient access to antibiotics (non-prescription use)

A systematic review published in 2011 looking at 35 community surveys from five continents showed that non-prescription antibiotic use occurred worldwide and accounted for 19–100% of antimicrobial use outside of northern Europe and North America [139]. Safety issues associated with non-prescription use included adverse drug reactions and masking of underlying infectious processes. Antimicrobial-resistant bacteria are common in communities with frequent non-prescription use, which has been speculated to play an important role in selecting and maintaining these high levels of community antimicrobial resistance. In middle-income to high-income countries with reliable access to healthcare practitioners, antimicrobials should be restricted to prescription-only status [139].

## Strengths and limitations

This review employed a comprehensive search strategy, encompassed over 20 years of publications and reviewed 139 relevant papers. The key findings from individual papers were analysed by the authors and integrated in a logical manner that is of use to front-line clinicians dealing with patients presenting with ARTI in primary care. Limitations were that only English language studies were included and that other indications for antibiotic prescription in primary care such as urinary tract infection were not considered. However, the commonest indication for antibiotic prescription in primary care is for ARTI. Patient expectation is a very important reason for PCPs to prescribe antibiotics, and it can change with good communication skills and patient-centred approach to the consultation. The most recent literature was felt to be of most relevance to practicing clinicians.

## Recommendations for further research

Future research should focus on developing and refining strategies that have been proven to be successful, such as multifaceted education of PCPs and the public, the use of delayed prescriptions and ensuring that consultations for ARTIs are not time pressurised. The use of less expensive resources such as non-medical practitioners as the first point of contact should be explored in the wider global context, especially in countries where there is direct access to antibiotics because of limited access to healthcare professionals. The role of other primary care resources such as community pharmacists and practice nurses should also be considered in the context of developing a more cohesive approach by all primary care health professionals who are consulted by people with ARTI. Coordination of policy led by the relevant postgraduate training bodies for these disciplines should be encouraged and developed in conjunction with the relevant state agencies. Studies should also focus on problem areas such as the attitudes of daycare providers and the difficulties of dealing with fee-paying patients. Also, in areas where the literature is contradictory such as with delayed prescriptions, further study is warranted. Further development and testing of POCTs to help reduce clinical uncertainty is advised. The importance of cultural and resource factors needs to be evaluated further, especially in the setting of direct patient access to antibiotics.

## Conclusion

Antimicrobial resistance is an important and growing health issue, and a considerable contributor is the overprescribing of antibiotics in primary care for ARTI.

Less pressurised consultations, training to enhance PCP communication skills, multifaceted education campaigns aimed at patients and PCPs, delayed prescriptions and point of care testing to reduce diagnostic uncertainty all help to reduce such inappropriate prescribing.

## References

[1] Aryee A, Price N (2014). Antimicrobial stewardship—can we afford to do without it?. Br J Clin Pharmacol.

[2] WHO. Antimicrobial Resistance. Global report on surveillance. Switzerland: 2014

[3] Bell BG, Schellevis F, Stobberingh E, Goossens H, Pringle M (2014). A systematic review and meta-analysis of the effects of antibiotic consumption on antibiotic resistance. BMC Infect Dis.

[4] Power RF, Linnane B, Martin R, Power N, Harnett P, Casserly B, O’Connell NH, Dunne CP (2016). The first reported case of Burkholderia contaminans in patients with cystic fibrosis in Ireland: from the Sargasso Sea to Irish Children. BMC Pulm Med.

[5] O'Connor C, Cormican M, Boo TW, McGrath E, Slevin B, O'Gorman A, Commane M, Mahony S, O'Donovan E, Powell J, Monahan R, Finnegan C, Kiernan MG, Coffey JC, Power L, O'Connell NH, Dunne CP (2016). An Irish outbreak of New Delhi metallo-beta-lactamase (NDM)-1 carbapenemase-producing Enterobacteriaceae: increasing but unrecognized prevalence. J Hosp Infect.

[6] O'Connor C, O'Connell NH, Commane M, O'Donovan E, Power L, Dunne CP (2016). Limerick: forever associated with five lines of rhyme or infamous for irrepressible carbapenemase-producing Enterobacteriaceae for all time?. J Hosp Infect.

[7] O’Connor C, Philip RK, Kelleher J, Powell J, O’Gorman A, Slevin B, Woodford N, Turton JF, McGrath E, Finnegan C, Power L, O’Connell NH, Dunne CP (2017). The first occurrence of a CTX-M ESBL-producing Escherichia coli outbreak mediated by mother to neonate transmission in an Irish neonatal intensive care unit. BMC Infect Dis.

[8] HPRA. Report on Antimicrobial Resistance. Report. Dublin: 2016 January. Report No

[9] Centre HPS (2017) Primary care antibiotic consumption rates

[10] Group SHASW (2009) Guidelines for antimicrobial stewardship in hospitals in Ireland

[11] HSE. Antibiotic Prescribing Dublin: HSE; 2017 [cited 2017 21/07/2017]. Available from: http://www.hse.ie/eng/services/list/2/gp/Antibiotic-Prescribing/Conditions-and-Treatments/List-of-Conditions-and-Treatments.html

[12] WHO. Antimicrobial resistance. Global Report on Surveillance Infographic 2014:1

[13] Nathan C, Cars O (2014). Antibiotic resistance—problems, progress, and prospects. N Engl J Med.

[14] Harbarth S, Balkhy HH, Goossens H, Jarlier V, Kluytmans J, Laxminarayan R (2015). Antimicrobial resistance: one world, one fight!. Antimicrob Resist Infect Control.

[15] NICE. Antimicrobial stewardship: systems and processes for effective antimicrobial medicine use United Kingdom: NICE; 2015 [cited 2017 22/6/17]. Available from: https://www.nice.org.uk/guidance/ng15/chapter/1-Recommendations

[16] NICE. Antimicrobial stewardship: changing risk related behaviours in the general population England, United Kingdom 2017 [cited 2017 22/6/17]. Available from: https://www.nice.org.uk/guidance/ng15/chapter/1-Recommendations

[17] Harris AM, Hicks LA, Qaseem A, for the High Value Care Task Force of the American College of P, for the Centers for Disease C, Prevention (2016). Appropriate antibiotic use for acute respiratory tract infection in adults: advice for high-value care from the American College of Physicians and the Centers for Disease Control and Prevention. Ann Intern Med.

[18] NICE. Self-limiting respiratory tract infections—antibiotic prescribing overview: United Kingdom; 2008 [cited 2017 22/6/17]. Available from: http://pathways.nice.org.uk/pathways/self-limiting-respiratory-tract-infections---antibiotic-prescribing

[19] Meropol SB, Localio AR, Metlay JP (2013). Risks and benefits associated with antibiotic use for acute respiratory infections: a cohort study. Ann Fam Med.

[20] Fahey T, Stocks N, Thomas T (1998). Systematic review of the treatment of upper respiratory tract infection. Arch Dis Child.

[21] Little P, Watson L, Morgan S, Williamson I (2002). Antibiotic prescribing and admissions with major suppurative complications of respiratory tract infections: a data linkage study. Br J Gen Pract.

[22] Butler CC, Hood K, Verheij T, Little P, Melbye H, Nuttall J, Kelly MJ, Molstad S, Godycki-Cwirko M, Almirall J, Torres A, Gillespie D, Rautakorpi U, Coenen S, Goossens H (2009). Variation in antibiotic prescribing and its impact on recovery in patients with acute cough in primary care: prospective study in 13 countries. BMJ.

[23] Gulliford MC, Moore MV, Little P, Hay AD, Fox R, Prevost AT (2016). Safety of reduced antibiotic prescribing for self limiting respiratory tract infections in primary care: cohort study using electronic health records. BMJ.

[24] Garbutt JM, Banister C, Spitznagel E, Piccirillo JF (2012). Amoxicillin for acute rhinosinusitis: a randomized controlled trial. JAMA.

[25] NICE. Acute cough—public expectation of symptom duration differs from published evidence United Kingdom: NICE; 2013 [cited 2017 22/6/17]. Available from: https://www.evidence.nhs.uk/search?q=%27Acute+cough+%E2%80%93+public+expectation+of+symptom+duration+differs+from+published+evidence%27

[26] Spinks A, Glasziou PP, Del Mar CB (2013) Antibiotics for sore throat. Cochrane Database Syst Rev (11):Cd000023 10.1002/14651858.CD000023.pub4 PMC6457983 24190439

[27] Little P, Gould C, Williamson I, Warner G, Gantley M, Kinmonth AL (1997). Reattendance and complications in a randomised trial of prescribing strategies for sore throat: the medicalising effect of prescribing antibiotics. BMJ.

[28] Yaeger JP, Temte JL, Hanrahan LP, Martinez-Donate P (2015). Roles of clinician, patient, and community characteristics in the management of pediatric upper respiratory tract infections. Ann Fam Med.

[29] Silverman M, Povitz M, Sontrop JM, Li L, Richard L, Cejic S, Shariff SZ (2017). Antibiotic prescribing for nonbacterial acute upper respiratory infections in elderly persons. Ann Intern Med.

[30] Schroeck JL, Ruh CA, Sellick JA, Ott MC, Mattappallil A, Mergenhagen KA (2015). Factors associated with antibiotic misuse in outpatient treatment for upper respiratory tract infections. Antimicrob Agents Chemother.

[31] Teng CL (2014). Antibiotic prescribing for upper respiratory tract infections in the Asia-Pacific region: a brief review. Malays Fam Physician : Off J Acad Fam Physicians Malaysia.

[32] Murphy M, Bradley CP, Byrne S (2012). Antibiotic prescribing in primary care, adherence to guidelines and unnecessary prescribing—an Irish perspective. BMC Fam Pract.

[33] Rún Sigurðardóttir N, Nielsen ABS, Munck A, Bjerrum L (2015). Appropriateness of antibiotic prescribing for upper respiratory tract infections in general practice: comparison between Denmark and Iceland. Scand J Prim Health Care.

[34] England PH (2015) Guidance. Health Matters: Antimicrob Resist

[35] Zuckerman IH, Perencevich EN, Harris AD (2007). Concurrent acute illness and comorbid conditions poorly predict antibiotic use in upper respiratory tract infections: a cross-sectional analysis. BMC Infect Dis.

[36] Fletcher-Lartey S, Yee M, Gaarslev C, Khan R (2016). Why do general practitioners prescribe antibiotics for upper respiratory tract infections to meet patient expectations: a mixed methods study. BMJ Open.

[37] Simpson SA, Wood F, Butler CC (2007). General practitioners’ perceptions of antimicrobial resistance: a qualitative study. J Antimicrob Chemother.

[38] Kumar S, Little P, Britten N (2003). Why do general practitioners prescribe antibiotics for sore throat? Grounded theory interview study. BMJ.

[39] Paluck E, Katzenstein D, Frankish CJ, Herbert CP, Milner R, Speert D, Chambers K (2001). Prescribing practices and attitudes toward giving children antibiotics. Can Fam Physician Medecin de famille canadien.

[40] Md Rezal RS, Hassali MA, Alrasheedy AA, Saleem F, Md Yusof FA, Godman B (2015). Physicians’ knowledge, perceptions and behaviour towards antibiotic prescribing: a systematic review of the literature. Expert Rev Anti-Infect Ther.

[41] Wong CK, Liu Z, Butler CC, Wong SY, Fung A, Chan D (2016). Help-seeking and antibiotic prescribing for acute cough in a Chinese primary care population: a prospective multicentre observational study. NPJ Prim Care Respir Med.

[42] Ebell MH, Lundgren J, Youngpairoj S (2013). How long does a cough last? Comparing patients’ expectations with data from a systematic review of the literature. Ann Fam Med.

[43] Gambarelli L, Montari C, Manni A, Bertolani U, Corti N, Pedroni M (2002) Antibiotics in viral upper respiratory tract infections Ricerca e Pratica ;18(4):152–6

[44] Biezen R, Brijnath B, Grando D, Mazza D (2017). Management of respiratory tract infections in young children—a qualitative study of primary care providers’ perspectives. NPJ Prim Care Respir Med.

[45] Lindberg Bent H., Gjelstad Svein, Foshaug Mats, Høye Sigurd (2017). Antibiotic prescribing for acute respiratory tract infections in Norwegian primary care out-of-hours service. Scandinavian Journal of Primary Health Care.

[46] Hamm RM, Hicks RJ, Bemben DA (1996). Antibiotics and respiratory infections: are patients more satisfied when expectations are met?. J Fam Pract.

[47] Shlomo V, Adi R, Eliezer K (2003) The knowledge and expectations of parents about the role of antibiotic treatment in upper respiratory tract infection—a survey among parents attending the primary physician with their sick child. BMC Fam Pract 4(1471–2296 (Electronic)) 10.1186/1471-2296-4-20 PMC321647 14700470

[48] Martin CL, Njike VY, Katz DL (2004). Back-up antibiotic prescriptions could reduce unnecessary antibiotic use in rhinosinusitis. J Clin Epidemiol.

[49] Soma M, Slapgard H, Lerberg M, Lindbaek M (2005). Patients’ expectations of antibiotics for acute respiratory tract infections. Tidsskrift for den Norske laegeforening : tidsskrift for praktisk medicin, ny raekke.

[50] Ong S, Nakase J, Moran GJ, Karras DJ, Kuehnert MJ, Talan DA (2007). Antibiotic use for emergency department patients with upper respiratory infections: prescribing practices, patient expectations, and patient satisfaction. Ann Emerg Med.

[51] Ackerman SL, Gonzales R, Stahl MS, Metlay JP (2013). One size does not fit all: evaluating an intervention to reduce antibiotic prescribing for acute bronchitis. BMC Health Serv Res.

[52] Hardy-Holbrook RA, Svetlana, Chandnani V, DeWindt D, Dinh K (2013). Antibiotic resistance and prescribing in Australia: current attitudes and practice of GPs. Healthcare Infect.

[53] McNulty CA, Nichols T, French DP, Joshi P, Butler CC (2013) Expectations for consultations and antibiotics for respiratory tract infection in primary care: the RTI clinical iceberg. BJGP (1478–5242 (Electronic)):e429-e36 10.3399/bjgp13X669149 PMC3693799 23834879

[54] Dempsey PP, Businger AC, Whaley LE, Gagne JJ, Linder JA (2014). Primary care clinicians' perceptions about antibiotic prescribing for acute bronchitis: a qualitative study. BMC Fam Pract.

[55] McKay R, Mah A, Law MR (2016) Systematic review of factors associated with antibiotic prescribing for respiratory tract infections. 60(7):4106–18 10.1128/AAC.00209-16 PMC4914667 27139474

[56] Patel A, Chaitoff A, Rothberg M (2017) Understanding physician treatment decisions for the management of upper respiratory tract infections. J Gen Intern Med 32 10.1007/s11606-019-05433-5 PMC7174444 31630364

[57] Sun BZ, Chaitoff A, Hu B, Neuendorf K, Manne M, Rothberg MB. Empathy, burnout, and antibiotic prescribing for acute respiratory infections: a cross-sectional primary care study in the US. (1478–5242 (Electronic)) 10.3399/bjgp17X691901 PMC5519128 28717000

[58] Zyoud SH, Abu Taha A, Araj KF, Abahri IA, Sawalha AF, Sweileh WM (2015). Parental knowledge, attitudes and practices regarding antibiotic use for acute upper respiratory tract infections in children: a cross-sectional study in Palestine. BMC Pediatr.

[59] Panagakou SG, Spyridis N, Papaevangelou V, Theodoridou KM, Goutziana GP, Theodoridou MN (2011). Antibiotic use for upper respiratory tract infections in children: a cross-sectional survey of knowledge, attitudes, and practices (KAP) of parents in Greece. BMC Pediatr.

[60] Vanden Eng J, Marcus R, Hadler JL, Imhoff B, Vugia DJ, Cieslak PR (2003). Consumer attitudes and use of antibiotics. Emerg Infect Dis.

[61] Gaarslev C, Yee M, Chan G, Fletcher-Lartey S, Khan R (2016). A mixed methods study to understand patient expectations for antibiotics for an upper respiratory tract infection. Antimicrob Resist Infect Control.

[62] Ritchie SR, Jayanatha KJ, Duffy EJ, Chancellor J, Allport Z, Thomas MG (2017). Previous antibiotic-related adverse drug reactions do not reduce expectations for antibiotic treatment of upper respiratory tract infections. J Glob Antimicrob Resist.

[63] Mohan S, Sisler K, Christopher K, Hentzelman J, Antisdel J (2014). Societal and physician perspectives on sinonasal diagnosis and treatment. Am J Rhinol Allergy.

[64] Haltiwanger KA, Hayden GF, Weber T, Evans BA, Possner AB (2001). Antibiotic-seeking behavior in college students: what do they really expect?. J Am Coll Health : J ACH.

[65] Ashworth M, White P, Jongsma H, Schofield P, Armstrong D (2016). Antibiotic prescribing and patient satisfaction in primary care in England: cross-sectional analysis of national patient survey data and prescribing data. Br J Gen Pract.

[66] Coenen S, Francis N, Kelly M, Hood K, Nuttall J, Little P, Verheij TJM, Melbye H, Goossens H, Butler CC, on behalf of the GRACE Project Group (2013). Are patient views about antibiotics related to clinician perceptions, management and outcome? A multi-country study in outpatients with acute cough. PLoS One.

[67] Courtenay M, Rowbotham S, Lim R, Deslandes R, Hodson K, MacLure K, Peters S, Stewart D (2017). Antibiotics for acute respiratory tract infections: a mixed-methods study of patient experiences of non-medical prescriber management. BMJ Open.

[68] Welschen I, Kuyvenhoven M, Hoes A, Verheij T (2004). Antibiotics for acute respiratory tract symptoms: patients’ expectations, GPs’ management and patient satisfaction. Fam Pract.

[69] Altiner A, Knauf A, Moebes J, Sielk M, Wilm S (2004). Acute cough: a qualitative analysis of how GPs manage the consultation when patients explicitly or implicitly expect antibiotic prescriptions. Fam Pract.

[70] Linder JA, Singer DE (2003). Desire for antibiotics and antibiotic prescribing for adults with upper respiratory tract infections. J Gen Intern Med.

[71] Kautz-Freimuth S, Redaèlli M, Samel C, Civello D, Altin SV, Stock S (2015). Parental views on acute otitis media (AOM) and its therapy in children—results of an exploratory survey in German childcare facilities. BMC Pediatr.

[72] Kallestrup P, Bro F (2003). Parents’ beliefs and expectations when presenting with a febrile child at an out-of-hours general practice clinic. Br J Gen Pract.

[73] Faber MS, Heckenbach K, Velasco E, Eckmanns T (2010) Antibiotics for the common cold: expectations of Germany’s general population. Euro Surveillance : bulletin Europeen sur les maladies transmissibles = European communicable disease bulletin 15(35) 20822733

[74] Tonkin-Crine S, Yardley L, Little P (2011). Antibiotic prescribing for acute respiratory tract infections in primary care: a systematic review and meta-ethnography. J Antimicrob Chemother.

[75] Llor C, Bjerrum L, Strandberg EL, Radzeviciene R, Reutskiy A, Caballero L (2013). Low request of antibiotics from patients with respiratory tract infections in six countries: results from the Happy Audit Study. Antibiotics (Basel, Switzerland).

[76] Strandberg EL, Brorsson A, Andre M, Grondal H, Molstad S, Hedin K (2016). Interacting factors associated with low antibiotic prescribing for respiratory tract infections in primary health care—a mixed methods study in Sweden. BMC Fam Pract.

[77] Vaz LE, Kleinman KP, Lakoma MD, Dutta-Linn MM, Nahill C, Hellinger J, Finkelstein JA (2015). Prevalence of parental misconceptions about antibiotic use. Pediatrics.

[78] Rousounidis A, Papaevangelou V, Hadjipanayis A, Panagakou S, Theodoridou M, Syrogiannopoulos G (2011). Descriptive study on parents’ knowledge, attitudes and practices on antibiotic use and misuse in children with upper respiratory tract infections in Cyprus. Int J Environ Res Public Health.

[79] Salazar ML, English TM, Eiland LS (2012). Caregivers’ baseline understanding and expectations of antibiotic use for their children. Clin Pediatr (Phila).

[80] Murphy Marion, Byrne Stephen, Bradley Colin P (2011). Influence of patient payment on antibiotic prescribing in Irish general practice: a cohort study. British Journal of General Practice.

[81] Ab Rahman N, Teng CL, Sivasampu S (2016). Antibiotic prescribing in public and private practice: a cross-sectional study in primary care clinics in Malaysia. BMC Infect Dis.

[82] Goossens H, Guillemot D, Ferech M, Schlemmer B, Costers M, van Breda M, Baker LJ, Cars O, Davey PG (2006). National campaigns to improve antibiotic use. Eur J Clin Pharmacol.

[83] Sabuncu E, David J, Bernede-Bauduin C, Pepin S, Leroy M, Boelle PY (2009). Significant reduction of antibiotic use in the community after a nationwide campaign in France, 2002-2007. PLoS Med.

[84] Lambert MF, Masters GA, Brent SL (2007). Can mass media campaigns change antimicrobial prescribing? A regional evaluation study. J Antimicrob Chemother.

[85] Grover ML, Nordrum JT, Mookadam M, Engle RL, Moats CC, Noble BN (2013). Addressing antibiotic use for acute respiratory tract infections in an academic family medicine practice. Am J Medical Qual : Off J Am Coll Med Qual.

[86] Huttner B, Goossens H, Verheij T, Harbarth S (2010). Characteristics and outcomes of public campaigns aimed at improving the use of antibiotics in outpatients in high-income countries. Lancet Infect Dis.

[87] Gonzales R, Sauaia A, Corbett KK, Maselli JH, Erbacher K, Leeman-Castillo BA (2004). Antibiotic treatment of acute respiratory tract infections in the elderly: effect of a multidimensional educational intervention. J Am Geriatr Soc.

[88] Taylor JA, Kwan-Gett TS, McMahon EM (2005). Effectiveness of a parental educational intervention in reducing antibiotic use in children: a randomized controlled trial. Pediatr Infect Dis J.

[89] Vodicka TA, Thompson M, Lucas P, Heneghan C, Blair PS, Buckley DI, Redmond N, Hay AD, TARGET Programme team (2013). Reducing antibiotic prescribing for children with respiratory tract infections in primary care: a systematic review. Br J Gen Pract.

[90] Gjelstad S, Hoye S, Straand J, Brekke M, Dalen I, Lindbaek M (2013). Improving antibiotic prescribing in acute respiratory tract infections: cluster randomised trial from Norwegian general practice (prescription peer academic detailing (Rx-PAD) study). BMJ.

[91] Vinnard C, Linkin DR, Localio AR, Leonard CE, Teal VL, Fishman NO, Hennessy S (2013). Effectiveness of interventions in reducing antibiotic use for upper respiratory infections in ambulatory care practices. Popul Health Manag.

[92] Little P, Stuart B, Francis N, Douglas E, Tonkin-Crine S, Anthierens S, Cals JW, Melbye H, Santer M, Moore M, Coenen S, Butler C, Hood K, Kelly M, Godycki-Cwirko M, Mierzecki A, Torres A, Llor C, Davies M, Mullee M, O'Reilly G, van der Velden A, Geraghty AW, Goossens H, Verheij T, Yardley L, GRACE consortium (2013). Effects of internet-based training on antibiotic prescribing rates for acute respiratory-tract infections: a multinational, cluster, randomised, factorial, controlled trial. Lancet.

[93] Ryves R, Eyles C, Moore M, McDermott L, Little P, Leydon GM (2016). Understanding the delayed prescribing of antibiotics for respiratory tract infection in primary care: a qualitative analysis. BMJ Open.

[94] Zeng L, Zhang L, Hu Z, Ehle EA, Chen Y, Liu L, Chen M (2014). Systematic review of evidence-based guidelines on medication therapy for upper respiratory tract infection in children with AGREE instrument. PLoS One.

[95] Elias C, Moja L, Mertz D, Loeb M, Forte G, Magrini N (2017). Guideline recommendations and antimicrobial resistance: the need for a change. BMJ Open.

[96] Harris RH, MacKenzie TD, Leeman-Castillo B, Corbett KK, Batal HA, Maselli JH (2003). Optimizing antibiotic prescribing for acute respiratory tract infections in an urban urgent care clinic. J Gen Intern Med.

[97] McKay RM, Vrbova L, Fuertes E, Chong M, David S, Dreher K, Purych D, Blondel-Hill E, Henry B, Marra F, Kendall PR, Patrick DM (2011). Evaluation of the do bugs need drugs? Program in British Columbia: can we curb antibiotic prescribing?. Can J Infect Dis Med Microbiol.

[98] Chaintarli K, Ingle SM, Bhattacharya A, Ashiru-Oredope D, Oliver I, Gobin M (2016). Impact of a United Kingdom-wide campaign to tackle antimicrobial resistance on self-reported knowledge and behaviour change. BMC Public Health.

[99] Hu Y, Walley J, Chou R, Tucker JD., Harwell JI, Wu X, Yin J, Zou G, Wei X (2016) Interventions to reduce childhood antibiotic prescribing for upper respiratory infections: systematic review and meta-analysis. J Epidemiol Community Health 10.1136/jech-2015-206543 27325869

[100] Dowell J, Pitkethly M, Bain J, Martin S (2001). A randomised controlled trial of delayed antibiotic prescribing as a strategy for managing uncomplicated respiratory tract infection in primary care. Br J Gen Pract.

[101] Arroll B, Kenealy T, Kerse N (2002). Do delayed prescriptions reduce the use of antibiotics for the common cold? A single-blind controlled trial. J Fam Pract.

[102] Edwards M, Dennison J, Sedgwick P (2003). Patients’ responses to delayed antibiotic prescription for acute upper respiratory tract infections. Br J Gen Pract.

[103] Little P, Rumsby K, Kelly J, Watson L, Moore M, Warner G, Fahey T, Williamson I (2005). Information leaflet and antibiotic prescribing strategies for acute lower respiratory tract infection: a randomized controlled trial. JAMA.

[104] Spurling GK, Del Mar CB, Dooley L, Foxlee R (2007) Delayed antibiotics for respiratory infections. (1469-493X (Electronic)) 10.1002/14651858.CD004417.pub3 17636757

[105] Moore M, Little P, Rumsby K, Kelly J, Watson L, Warner G, Fahey T, Williamson I (2009). Effect of antibiotic prescribing strategies and an information leaflet on longer-term reconsultation for acute lower respiratory tract infection. Br J Gen Pract.

[106] Agnew J, Taaffe M, Darker C, O'Shea B, Clarke J (2013). Delayed prescribing of antibiotics for respiratory tract infections: use of information leaflets. Ir Med J.

[107] Little P, Moore M, Kelly J, Williamson I, Leydon G, McDermott L, Mullee M, Stuart B, On behalf of the PIPS Investigators (2014). Delayed antibiotic prescribing strategies for respiratory tract infections in primary care: pragmatic, factorial, randomised controlled trial. BMJ.

[108] de la Poza Abad M, Mas Dalmau G, Moreno Bakedano M, Gonzalez Gonzalez AI, Canellas Criado Y, Hernandez Anadon S (2016). Prescription strategies in acute uncomplicated respiratory infections: a randomized clinical trial. JAMA Intern Med.

[109] Moore M, Stuart B, Hobbs FR, Butler CC, Hay AD, Campbell J (2017). Symptom response to antibiotic prescribing strategies in acute sore throat in adults: the DESCARTE prospective cohort study in UK general practice. Br J Gen Pract.

[110] Moore M, Stuart B, Hobbs FR, Butler CC, Hay AD, Campbell J (2017). Influence of the duration of penicillin prescriptions on outcomes for acute sore throat in adults: the DESCARTE prospective cohort study in UK general practice. Br J Gen Pract.

[111] Francis NA, Gillespie D, Nuttall J, Hood K, Little P, Verheij T, Goossens H, Coenen S, Butler CC (2012). Delayed antibiotic prescribing and associated antibiotic consumption in adults with acute cough. Br J Gen Pract.

[112] Peters S, Rowbotham S, Chisholm A, Wearden A, Moschogianis S, Cordingley L, Baker D, Hyde C, Chew-Graham C (2011). Managing self-limiting respiratory tract infections: a qualitative study of the usefulness of the delayed prescribing strategy. Br J Gen Pract.

[113] Hoye S, Frich JC, Lindbaek M. Use and feasibility of delayed prescribing for respiratory tract infections: a questionnaire survey. (1471–2296 (Electronic)) 10.1186/1471-2296-12-34 PMC3114766 21592334

[114] McDermott L, Leydon GM, Halls A, Kelly J, Nagle A, White J et al (2017) Qualitative interview study of antibiotics and self-management strategies for respiratory infections in primary care. BMJ Open 7(11) 10.1136/bmjopen-2017-016903 PMC5719297 29180593

[115] Høye Sigurd, Frich Jan, Lindbœk Morten (2010). Delayed prescribing for upper respiratory tract infections: a qualitative study of GPs' views and experiences. British Journal of General Practice.

[116] Francis NA, Butler CC, Hood K, Simpson S, Wood F, Nuttall J (2009). Effect of using an interactive booklet about childhood respiratory tract infections in primary care consultations on reconsulting and antibiotic prescribing: a cluster randomised controlled trial. BMJ.

[117] Francis NA, Phillips R, Wood F, Hood K, Simpson S, Butler CC (2013). Parents’ and clinicians’ views of an interactive booklet about respiratory tract infections in children: a qualitative process evaluation of the EQUIP randomised controlled trial. BMC Fam Pract.

[118] O'Sullivan JW, Harvey RT, Glasziou PP, McCullough A (2016) Written information for patients (or parents of child patients) to reduce the use of antibiotics for acute upper respiratory tract infections in primary care. CDSR (1469-493X (Electronic)) 10.1002/14651858.CD011360.pub2 PMC6464519 27886368

[119] Anthierens S, Tonkin-Crine S, Cals JW, Coenen S, Yardley L, Brookes-Howell L (2015). Clinicians’ views and experiences of interventions to enhance the quality of antibiotic prescribing for acute respiratory tract infections. J Gen Intern Med.

[120] Filipetto FA, Modi DS, Weiss LB, Ciervo CA (2008). Patient knowledge and perception of upper respiratory infections, antibiotic indications and resistance. Patient Preference Adherence.

[121] Little P, Hobbs FDR, Moore M, Mant D, Williamson I, McNulty C, Cheng YE, Leydon G, McManus R, Kelly J, Barnett J, Glasziou P, Mullee M, on behalf of the PRISM investigators (2013) Clinical score and rapid antigen detection test to guide antibiotic use for sore throats: randomised controlled trial of PRISM (primary care streptococcal management). BMJ. Br Med J 347 10.1136/bmj.f5806 PMC3805475 24114306

[122] Mangione-Smith R, Zhou C, Robinson JD, Taylor JA, Elliott MN, Heritage J (2015). Communication practices and antibiotic use for acute respiratory tract infections in children. Ann Fam Med.

[123] Cabral C, Ingram J, Lucas PJ, Redmond NM, Kai J, Hay AD, Horwood J (2016). Influence of clinical communication on parents’ antibiotic expectations for children with respiratory tract infections. Ann Fam Med.

[124] Brookes-Howell Lucy, Hood Kerenza, Cooper Lucy, Little Paul, Verheij Theo, Coenen Samuel, Godycki-Cwirko Maciek, Melbye Hasse, Borras-Santos Alicia, Worby Patricia, Jakobsen Kristin, Goossens Herman, Butler Christopher C (2012). Understanding variation in primary medical care: a nine-country qualitative study of clinicians’ accounts of the non-clinical factors that shape antibiotic prescribing decisions for lower respiratory tract infection. BMJ Open.

[125] Cabral C, Horwood J, Hay AD, Lucas PJ (2014). How communication affects prescription decisions in consultations for acute illness in children: a systematic review and meta-ethnography. BMC Fam Pract.

[126] Coxeter P, Del Mar CB, McGregor L, Beller EM, Hoffmann TC (2015) Interventions to facilitate shared decision making to address antibiotic use for acute respiratory infections in primary care. Cochrane Database Syst Rev (11):Cd010907 10.1002/14651858.CD010907.pub2 PMC6464273 26560888

[127] Tonkin-Crine SK, Tan PS, van Hecke O, Wang K, Roberts NW, McCullough A (2017). Clinician-targeted interventions to influence antibiotic prescribing behaviour for acute respiratory infections in primary care: an overview of systematic reviews. Cochrane Database Syst Rev.

[128] Lee SS, Yau B, Huang JQ, You JHS (2008). Determinants of appropriate antibiotic use in the community—a survey in Sydney and Hong Kong. Br J Infect Control.

[129] Fischer T, Fischer S, Kochen MM, Hummers-Pradier E (2005). Influence of patient symptoms and physical findings on general practitioners’ treatment of respiratory tract infections: a direct observation study. BMC Fam Pract.

[130] Wigton RS, Darr CA, Corbett KK, Nickol DR, Gonzales R (2008). How do Community practitioners decide whether to prescribe antibiotics for acute respiratory tract infections?. J Gen Intern Med.

[131] O'Brien K, Bellis TW, Kelson M, Hood K, Butler CC, Edwards A. Clinical predictors of antibiotic prescribing for acutely ill children in primary care: an observational study. (1478–5242 (Electronic)) 10.3399/bjgp15X686497 PMC4540398 26324495

[132] Mehta N, Schilder A, Fragaszy E, E R Evans H, Dukes O, Manikam L et al (2017) Antibiotic prescribing in patients with self-reported sore throat. (1460–2091 (Electronic)) 10.1093/jac/dkw497 PMC5400092 27999063

[133] Rebnord IK, Sandvik H, Mjelle AB, Hunskaar S (2017). Factors predicting antibiotic prescription and referral to hospital for children with respiratory symptoms: secondary analysis of a randomised controlled study at out-of-hours services in primary care. BMJ Open.

[134] Kavanagh KE, O'Shea E, Halloran R, Cantillon P, Murphy AW (2011) A pilot study of the use of near-patient C-reactive protein testing in the treatment of adult respiratory tract infections in one Irish general practice. BMC Fam Pract. (1471–2296 (Electronic)) 10.1186/1471-2296-12-93 PMC3175160 21880122

[135] Wood F, Brookes-Howell L, Hood K, Cooper L, Verheij T, Goossens H, Little P, Godycki-Cwirko M, Adriaenssens N, Jakobsen K, Butler CC (2011). A multi-country qualitative study of clinicians’ and patients’ views on point of care tests for lower respiratory tract infection. Fam Pract.

[136] Leydon GM, McDermott L, Moore M, Williamson I, Hobbs FD, Lambton T, Cooper R, Henderson H, Little P, PRISM Investigators (2013). A qualitative study of GP, NP and patient views about the use of rapid streptococcal antigen detection tests (RADTs) in primary care: ‘swamped with sore throats’?. BMJ Open.

[137] Debets VE, Verheij TJ, van der Velden AW (2017). Antibiotic prescribing during office hours and out-of-hours: a comparison of quality and quantity in primary care in the Netherlands. Br J Gen Pract.

[138] Rooshenas L, Wood F, Brookes-Howell L, Evans MR, Butler CC (2014). The influence of children’s day care on antibiotic seeking: a mixed methods study. Br J Gen Pract.

[139] Morgan DJ, Okeke IN, Laxminarayan R, Perencevich EN, Weisenberg S (2011). Non-prescription antimicrobial use worldwide: a systematic review. Lancet Infect Dis.

